# *Legionella* Eukaryotic-Like Type IV Substrates Interfere with Organelle Trafficking

**DOI:** 10.1371/journal.ppat.1000117

**Published:** 2008-08-01

**Authors:** Karim Suwwan de Felipe, Robert T. Glover, Xavier Charpentier, O. Roger Anderson, Moraima Reyes, Christopher D. Pericone, Howard A. Shuman

**Affiliations:** 1 Integrated Program in Cellular, Molecular, and Biophysical Studies, Columbia University Medical Center, New York, New York, United States of America; 2 Department of Microbiology, Columbia University Medical Center, New York, New York, United States of America; 3 Division of Biology and Paleo Environment, Lamont-Doherty Earth Observatory, Columbia University, Palisades, New York, United States of America; Tufts University School of Medicine, United States of America

## Abstract

*Legionella pneumophila*, the causative agent of Legionnaires' disease, evades phago-lysosome fusion in mammalian and protozoan hosts to create a suitable niche for intracellular replication. To modulate vesicle trafficking pathways, *L. pneumophila* translocates effector proteins into eukaryotic cells through a Type IVB macro-molecular transport system called the Icm-Dot system. In this study, we employed a fluorescence-based translocation assay to show that 33 previously identified *Legionella* eukaryotic-like genes (*leg*) encode substrates of the Icm-Dot secretion system. To assess which of these proteins may contribute to the disruption of vesicle trafficking, we expressed each gene in yeast and looked for phenotypes related to vacuolar protein sorting. We found that LegC3-GFP and LegC7/YlfA-GFP caused the mis-secretion of CPY-Invertase, a fusion protein normally restricted to the yeast vacuole. We also found that LegC7/YlfA-GFP and its paralog LegC2/YlfB-GFP formed large structures around the yeast vacuole while LegC3-GFP localized to the plasma membrane and a fragmented vacuole. In mammalian cells, LegC2/YlfB-GFP and LegC7/YlfA-GFP were found within large structures that co-localized with anti-KDEL antibodies but excluded the lysosomal marker LAMP-1, similar to what is observed in Legionella-containing vacuoles. LegC3-GFP, in contrast, was observed as smaller structures which had no obvious co-localization with KDEL or LAMP-1. Finally, LegC3-GFP caused the accumulation of many endosome-like structures containing undigested material when expressed in the protozoan host *Dictyostelium discoideum*. Our results demonstrate that multiple Leg proteins are Icm/Dot-dependent substrates and that LegC3, LegC7/YlfA, and LegC2/YlfB may contribute to the intracellular trafficking of *L. pneumophila* by interfering with highly conserved pathways that modulate vesicle maturation.

## Author Summary

*Legionella pneumophila* is a Gram-negative bacterial species that causes a severe pneumonia known as Legionnaires' disease. Inhalation of *L. pneumophila*–contaminated aerosols results in the infection of lung macrophages. Following infection, the bacteria use a Type IVB secretion system to deliver multiple effector proteins into the macrophages to create a membrane-bound replicative compartment called the Legionella-containing vacuole, or LCV. The LCV is defined by its recruitment of early secretory vesicles and avoidance of the bactericidal lysosomes. We identified several effector proteins that contain eukaryotic domains and share significant homology with eukaryotic organelle trafficking proteins. We demonstrate that 33 *Legionella* eukaryotic-like genes (*leg*) encode proteins that are translocated into host cells. When artificially expressed in yeast, three Leg proteins (LegC2, LegC3, and LegC7) were able to disrupt normal vesicle trafficking and vacuole morphology. In addition, the Leg proteins induced the formation of, and were localized within, distinct structures when expressed in mammalian cells. In the protozoan host *Dictyostelium discoideum*, expression of LegC3 resulted in the accumulation of membranous compartments containing partially digested material. Thus, LegC3, LegC2, and LegC7 represent novel effector proteins that may contribute to the intracellular lifestyle of *L. pneumophila* by disrupting normal vacuolar trafficking pathways in host cells.

## Introduction

When inhaled by humans, the γ-proteobacterial species *Legionella pneumophila* can cause a severe, acute and often fatal form of pneumonia known as Legionnaires' disease [1]–[3]. *Legionella sp.* remain one of the leading causes of community-acquired pneumonia, with poor diagnosis and inadequate treatment accounting for many of the reported fatalities [4],[5]. *L. pneumophila* is an environmental organism often found replicating inside phylogenetically diverse species of amoeba and its ability to cause disease in humans is believed to be largely accidental [6]–[11]. Even though the specific requirements for replication inside protozoans and macrophages may differ [12],[13], *L. pneumophila's* lifestyle in each host is quite similar.

Wild type *L. pneumophila* first promotes its own phagocytosis [14]–[16] and then rapidly avoids phago-lysosome fusion [17]–[19]. The pathogen then forms a replicative vacuole rich in early secretory vesicles and ER-derived vesicles [20]–[24]. Effector proteins specifically delivered into host cells by the Icm-Dot Type IVB secretion system are believed to supplant or modify normal organelle trafficking to generate and sustain the Legionella-containing vacuole (LCV) [13],[25]. Several effector proteins have been identified which may be responsible for recruiting vesicles from the ER to the LCV. For example, the effector protein RalF recruits ADP-ribosylation factor -1, a critical regulator of ER and Golgi vesicle formation, to the LCV [26]. Another regulator of ER and Golgi vesicle traffic, Rab1, is both recruited to the LCV and activated by the effector proteins DrrA (SidM) and LidA [27]–[29]. Additional signaling molecules may be targets of *L. pneumophila* effectors as well, including phosphoinositides [30] and ubiquitinylated proteins [31].

Recently, multiple putative effector proteins have been identified via genetic, biochemical and bioinformatic screens [26], [32]–[44]. Interestingly, many *L. pneumophila* effector proteins have been found to contain eukaryotic domains or have overall similarity to eukaryotic proteins [26],[33],[43],[45],[46]. Interdomain horizontal gene transfer has been proposed as a mechanism through which these Legionella eukaryotic-like genes (*leg*) may have been acquired [26],[43],[45],[47]. Although the functions of some of these effectors have been elucidated, the great majority remain uncharacterized. With only a few exceptions, virulence phenotypes associated with genetic deletions of individual effector proteins have not been observed, presumably due to functional redundancy within the pool of translocated effectors [33],[34],[43] and/or host specificity [33],[39],[48]. Thus, because more than one effector protein may affect a single host protein or pathway, determining the significance of individual effectors has proven challenging.

Although non-phagocytic, *Saccharomyces cerevisiae* shares many of the same trafficking pathways of higher eukaryotes [49],[50], and thus offers an attractive model for the study of bacterial virulence factors. Effectors from many bacterial pathogens, including *Chlamydia, Shigella, Pseudomonas, Yersinia,* and *Salmonella* have been studied in yeast [51]–[54]. Furthermore, yeast models have already been used to identify and analyze *L. pneumophila* effectors based on their lethal effects [36] and their ability to disrupt normal vacuolar protein sorting (VPS) and early secretory machinery [22],[38].

In this work, we report the identification of three *L. pneumophila* effector proteins, LegC3, LegC2/YlfA, and LegC7/YlfB, which are sufficient to cause VPS defects and/or altered vacuolar morphology in yeast. Importantly, these proteins induce the formation of and are located within similar structures when expressed in mammalian cells. Co-localization evidence is provided supporting the hypothesis that LegC3-GFP, LegC2/YlfB-GFP and LegC7/YlfA-GFP are effector proteins of *L. pneumophila* that can modify the normal endolysosomal pathway.

## Results

### Icm-Dot Dependent Translocation of Leg Proteins into J774 Cells

In a previous report, we used a bio-informatic approach to identify *L. pneumophila* eukaryotic-like genes (*leg* genes) based on the occurrence of eukaryotic motifs [43]. In order to determine which Leg proteins are substrates of the Icm-Dot system, we utilized a novel reporter system previously used for Type-III effector proteins [55]. In this system, TEM1 (ß-lactamase) is fused to a putative effector protein and a strain containing this fusion protein is used to infect host cells. Host cells are then loaded with CCF4/AM which, when excited at 409 nm emits green fluorescence (520 nm) due to *f*luorescence *r*esonance *e*nergy *t*ransfer (FRET) between the coumarin and fluorescein fluorophores. If the fusion protein was translocated into host cells, it cleaves the ß-lactam ring of CCF4/AM, releasing the two fluorophores and changing the fluorescence emission from green to blue (447 nm) when excited at the same wavelength. The ratio of blue to green fluorescence can then be quantified using a spectrofluorimeter with the appropriate excitation and emission filters.

**Figure 1:**
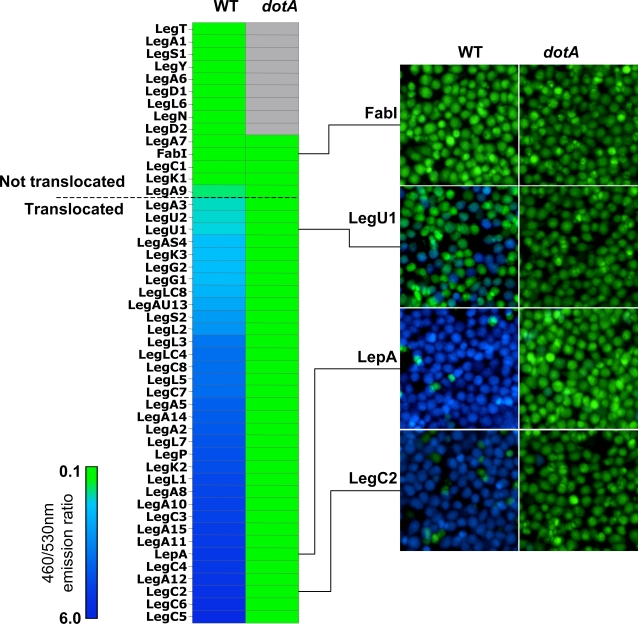
Icm-Dot Dependent Translocation Leg Substrates into J774 Cells. J774 cells were infected with KS79 (WT) or KS79*dotA* harboring TEM1-Leg protein fusions at an MOI of 50. Infected cells were loaded with CCF4/AM and translocation was determined by measuring the ratio of cleaved (460 nm) to uncleaved (530 nm) CCF4/AM. Ratios of 460/530 nm emission for each TEM1-Leg protein in wild type KS79 (WT) or KS79 *dotA* is shown as a heat diagram. A 460/530 nm ratio of more than 1 indicates translocation and is indicated by a horizontal dashed line. Representative images obtained using epifluorescence microscopy on individual assay wells is shown on the right. The results shown represent the average of 2 to 3 experiments, each performed in triplicate.

We previously found that 8 Leg-CyaA hybrid proteins out of 20 tested are substrates of the Icm-Dot system when CyaA was fused to the C-terminal end of the proteins [43]. In this study, we constructed TEM1 translational fusions to all 45 *leg* genes in which the Leg protein is fused to the C-terminus of TEM1 (Table 1). Using the fluorescence-based assay, we then tested if these TEM1-Leg hybrid proteins are translocated into J774 mouse macrophages. We consider a protein to be translocated if the ratio of blue to green fluorescence in host cells is greater than 1 after 60 minutes of contact with *L. pneumophila* expressing the hybrid constructs. If a ratio of less than one over background is observed in mutants that lack essential components of the translocon (*dotA* or *icmT*), we consider translocation to be dependent on the Icm-Dot system. We used a TEM1-FabI hybrid protein as a negative control, to show that the overexpression of a housekeeping protein (Enoyl-acyl CoA Reductase) does not result in non-specific translocation through the Icm-Dot secretion system. We also performed immunoblots using anti-ß-lactamase antibodies on *L. pneumophila* strains carrying all hybrid constructs, including those that were found not to be translocated. With the exception of TEM-LegA7, all hybrids are expressed at similar levels (data not shown). We found that 33 of the 45 Leg hybrid proteins tested are translocated in an Icm-Dot dependent manner (Figure 1). Importantly, 23 of these Leg proteins were not previously known to be translocated and 4 proteins (LegP, LegS2, LegC4 and LegA3) that had given no translocation signal using the C-terminal CyaA fusion system are in fact translocated. The 15 N-terminal TEM1 fusions found not to be translocated in this assay were also not translocated as C-terminal TEM1 fusions (data not shown). These results demonstrate that a large fraction (33/45) of the previously predicted Leg proteins are Icm-Dot substrates.

**Table 1:** Plasmids Used in This Study

Plasmids	Relevant Characteristics	References
pMMB207C	pMMB207 Δ*mobA*	[33]
pXDC61	pMMB207C *blaM*	This study
pXDC61-A1	pXDC61 *legA1*	This study
pXDC61-A2	pXDC61 *legA2*	This study
pXDC61-A3	pXDC61 *legA3*	This study
pXDC61-AS4	pXDC61 *legAS4*	This study
pXDC61-A6	pXDC61 *legA6*	This study
pXDC61-A7	pXDC61 *legA7*	This study
pXDC61-A8	pXDC61 *legA8*	This study
pXDC61-A9	pXDC61 *legA9*	This study
pXDC61-A10	pXDC61 *legA10*	This study
pXDC61-A11	pXDC61 *legA11*	This study
pXDC61-A12	pXDC61 *legA12*	This study
pXDC61-AU13	pXDC61 *legAU13*	This study
pXDC61-A14	pXDC61 *legA14*	This study
pXDC61-A15	pXDC61 *legA15*	This study
pXDC61-C1	pXDC61 *legC1*	This study
pXDC61-C2	pXDC61 *legC2*	This study
pXDC61-C3	pXDC61 *legC3*	This study
pXDC61-C4	pXDC61 *legC4*	This study
pXDC61-C5	pXDC61 *legC5*	This study
pXDC61-C6	pXDC61 *legC6*	This study
pXDC61-C7	pXDC61 *legC7*	This study
pXDC61-C8	pXDC61 *legC8*	This study
pXDC61-D1	pXDC61 *legD1*	This study
pXDC61-D2	pXDC61 *legD2*	This study
pXDC61-G1	pXDC61 *legG1*	This study
pXDC61-G2	pXDC61 *legG2*	This study
pXDC61-K1	pXDC61 *legK1*	This study
pXDC61-K2	pXDC61 *legK2*	This study
pXDC61-K3	pXDC61 *legK3*	This study
pXDC61-L1	pXDC61 *legL1*	This study
pXDC61-L2	pXDC61 *legL2*	This study
pXDC61-L3	pXDC61 *legL3*	This study
pXDC61-LC4	pXDC61 *legLC4*	This study
pXDC61-L5	pXDC61 *legL5*	This study
pXDC61-L6	pXDC61 *legL6*	This study
pXDC61-L7	pXDC61 *legL7*	This study
pXDC61-LC8	pXDC61 *legLC8*	This study
pXDC61-N	pXDC61 *legN*	This study
pXDC61-P	pXDC61 *legP*	This study
pXDC61-S1	pXDC61 *legS1*	This study
pXDC61-S2	pXDC61 *legS2*	This study
pXDC61-T	pXDC61 *legT*	This study
pXDC61-U1	pXDC61 *legU1*	This study
pXDC61-U2	pXDC61 *legU2*	This study
pXDC61-Y	pXDC61 *legY*	This study
pXDC61-LepA	pXDC61 *lepA*	This study
pXDC61-LepB	pXDC61 *lepB*	This study
pBM272	GAL inducible vector	[60]
pKS84	pBM272 *gfp*	This study
pKS99	pKS84 *legC1*	This study
pKS100	pKS84 *legC2*	This study
pKS101	pKS84 *legC3*	This study
pKS102	pKS84 *legC4*	This study
pKS103	pKS84 *legC5*	This study
pKS104	pKS84 *legC6*	This study
pKS105	pKS84 *legC7*	This study
pKS106	pKS84 *legC8*	This study
pKS107	pKS84 *legD1*	This study
pKS108	pKS84 *legD2*	This study
pKS109	pKS84 *legG1*	This study
pKS110	pKS84 *legG2*	This study
pKS111	pKS84 *legK1*	This study
pKS112	pKS84 *legK2*	This study
pKS113	pKS84 *legK3*	This study
pKS114	pKS84 *legL1*	This study
pKS115	pKS84 *legL2*	This study
pKS116	pKS84 *legL3*	This study
pKS117	pKS84 *legLC4*	This study
pKS118	pKS84 *legL5*	This study
pKS119	pKS84 *legL6*	This study
pKS120	pKS84 *legL7*	This study
pKS121	pKS84 *legLC8*	This study
pKS122	pKS84 *legN*	This study
pKS123	pKS84 *legP*	This study
pKS124	pKS84 *legS1*	This study
pKS125	pKS84 *legS2*	This study
pKS126	pKS84 *legT*	This study
pKS127	pKS84 *legU1*	This study
pKS128	pKS84 *legU2*	This study
pKS129	pKS84 *legY*	This study
pKS130	pKS84 *lepA*	This study
pKS131	pKS84 *lepB*	This study
pKS132	pBM272 *vps4EQ*	This study
pKS133	pBM272 *legC2*	This study
pKS134	pBM272 *legC3*	This study
pKS135	pBM272 *legC5*	This study
pKS136	pBM272 *legC7*	This study
pKS137	pBM272 *legC8*	This study
pMB35	Transactivator Plasmid	[75]
pMB38	Tet-off System Response Plasmid	[75]
pMB38-GFP	Tet-off System Response Plasmid containing GFP-S65T	Gift from B. Weissenmayer
pKS138	pMB38-GFP-His7x	This study
pKS140	pKS138 *legC3*	This study
pEGFP-N1	Mammalian expression vector	Clontech
pRG6	pEGFP-N1-legC3	This study
pRG7	pEGFP-N1-legC3ΔTM	This study
pRG8	pEGFP-N1-legC2	This study
pRG10	pEGFP-N1-legC7	This study

### LegC3 and LegC7/YlfA cause a Vacuolar Protein Sorting Defect in Yeast

One successful strategy used to identify proteins that are important for intracellular trafficking has been to search for gene-products that are required for *v*acuolar *p*rotein *s*orting (VPS) in yeast [56]–[58]. Many genes required for VPS are also important for endosomal trafficking and maturation in higher eukaryotes, including mammals [50]. Since the Legionella*-*containing phagosome avoids maturation and fusion to lysosomes, we hypothesized that *L. pneumophila* may translocate effectors that specifically interfere with conserved VPS pathways. In fact, it has been recently shown that 3 *L. pneumophila* effectors (VipA, VipD, VipF) interfere with VPS in yeast [38] while LidA interferes with steps in the secretory pathway [22].

**Figure 2:**
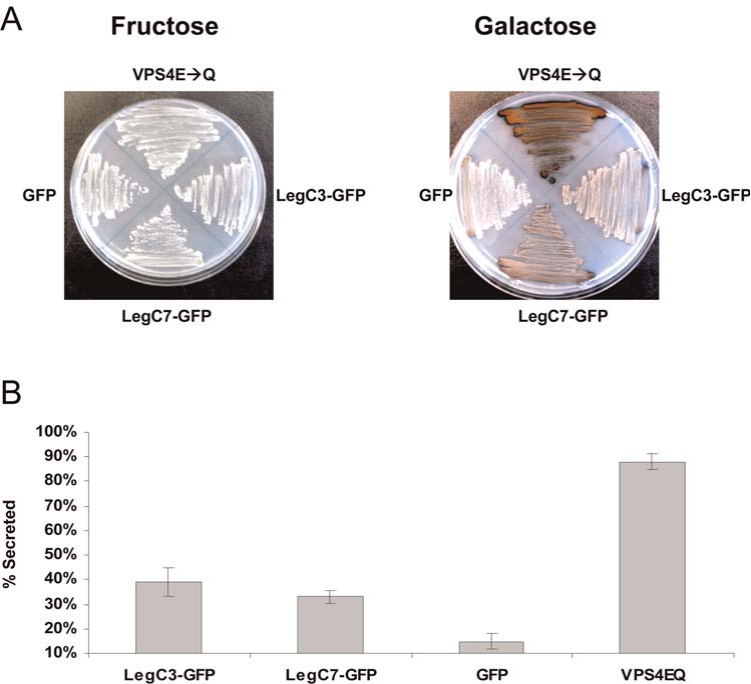
Effector-Induced Lethality in Yeast. Yeast cells containing expression vectors for LegC5-GFP, LegC8-GFP, LepB-GFP fusion proteins or GFP alone were spotted in 10-fold serial dilutions on SC-ura/fructose or SC-ura/galactose plates. Under conditions that induce expression of the hybrid protein (galactose), LegC5-GFP, LegC8-GFP and LepB-GFP impair yeast growth. Under non-inducing conditions (fructose), all strains grow comparably well.

To find out if any Leg proteins cause a VPS defect in yeast, we screened 29 strains that express the Leg proteins or LepA using an invertase overlay assay (Table 1). This assay detects mis-secreted CPY-Invertase, a protein which normally trafficks to the vacuole [59]. The plasmid used for sub-cloning, based on the low-copy pBM272 vector [60], creates C-terminal GFP translational fusions to the proteins of interest with transcription regulated by a galactose-inducible promoter. We found that the expression of LegC3-GFP and LegC7/YlfA-GFP caused the formation of a brown precipitate used to identify the aberrant presence of invertase activity on the surface of yeast colonies (Figure 2A). Strains expressing GFP alone did not cause a precipitate to form, whereas a strain expressing the Class E dominant negative protein VPS4^E233Q^ [61] induced a strong VPS defect. The qualitative assay was further verified using a quantitative assay (Figure 2B). We conclude from these results that the *L. pneumophila* effector proteins LegC3-GFP and LegC7/YlfA-GFP induce a VPS defect when expressed in yeast cells.

**Figure 3:**
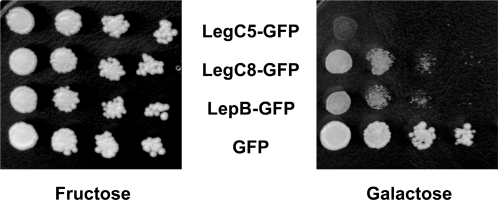
LegC3 and LegC7 Induce a Vacuolar Protein Sorting Defect in Yeast. (A) Yeast strains expressing CPY-Inv and LegC3-GFP, LegC7-GFP, GFP or VPS4^E233Q^ were streaked on SC-ura/fructose or SC-ura/galactose and grown for four days at 30°C. As described in the Materials and Methods, an agar solution containing chromogenic reagents to detect invertase activity was overlaid on the plates. The secretion of the CPY-Invertase hybrid is detected by the formation of a brown precipitate. (B) Quantitative assay for invertase secretion. Liquid cultures were grown to stationary phase and secreted versus total invertase activity was measured as described in Material and Methods. The results shown are of a representative experiment done in triplicate +/− standard deviations.

Galactose-induced expression of LegC5-GFP, LegC8-GFP, and the previously described LepB-GFP each caused a severe growth impairment in yeast cells (Figure 3) and conclusions regarding their effect on vacuolar sorting could not be drawn. This growth phenotype was not dependent on the presence of the GFP tag, since strains that expressed these Leg proteins or LepB in the absence of additional sequence exhibited similar growth defects (data not shown). Some Leg-GFP fusion proteins in this screen (LegC1, LegC4, LegD2, LegK1, LegN, and LegY) did not produce a fluorescent product as determined by epifluorescence microscopy and others (LegK2, LegK3, LegL2, LegLC4, LegL7, LegLC8, LegT, and LegU1) were not detectable by Western immunoblotting with anti-GFP antibody (Figure S1). These proteins may be poorly translated or be highly unstable when expressed in yeast cells. Of the fusion proteins where no VPS defect was detected, a considerable amount of heterogeneity with respect to protein levels was also observed. Similar to the toxic gene products LegC5, LegC8 and LepB, it remains possible that some of the poorly expressed Leg proteins may disrupt vacuolar sorting or cause toxicity if different expression systems are used.

### Intracellular Localization of Leg Proteins in Yeast and their Effects on Endocytic Trafficking to the Vacuole

The styryl dye FM4-64 is commonly used to monitor the trafficking of endocytic intermediates to the vacuole in yeast [62]. The dye first labels the plasma membrane, followed by labeling of internalized membrane, endocytic intermediates and finally the vacuole. Cells that have a VPS defect may present vacuolar abnormalities that are organized into 6 distinct classes [63]. These abnormalities can vary substantially and include defects in acidification, vacuolar fragmentation, and accumulation of endocytic vesicles in the pre-vacuole compartment.

**Figure 4:**
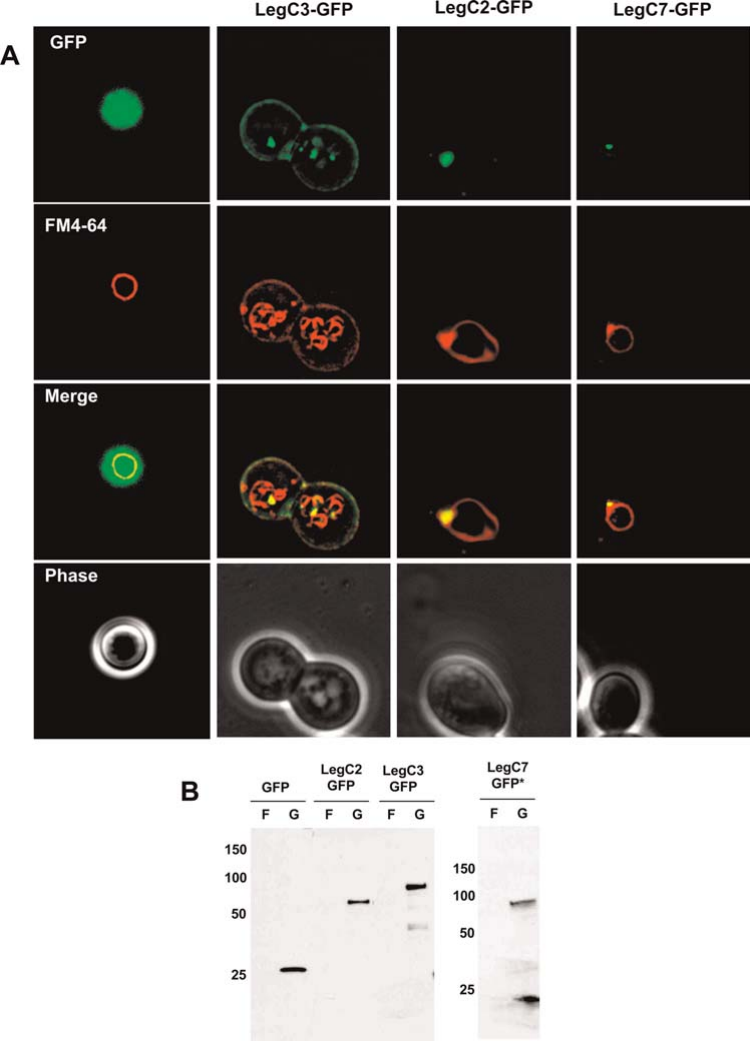
Steady State Localization of Leg-GFP Hybrids and Vacuole Defects. (A) Yeast strains expressing the LegC2-GFP, LegC3-GFP, LegC7-GFP hybrid proteins and GFP alone were harvested and pulse-chased with N-(3-triethylammoniumpropyl)-4-(p-diethylaminophenylhexatrienyl)-pyridinium dibromide (FM4-64) for vacuole visualization. Cells were viewed using epifluorescence microscopy. The images shown are representative of the overall population of cells expressing the Leg-GFP hybrids. (B) Whole cell lysate immunoblot using a rabbit polyclonal antibody against GFP showing that all the GFP Leg fusion proteins are expressed under galactose induction, but not when grown in fructose. The asterisk (*) is placed to emphasize that ten times more LegC7-GFP sample had to be loaded for visualization.

Fluorescence microscopy was used to visualize the intracellular localization of 30 Leg-GFP protein hybrids with the aid of FM4-64 to examine the vacuolar membrane. Most Leg-GFP proteins appeared to be cytosolic or were not easily detectable and had no apparent effect on the labeling of the vacuole by FM4-64 (data not shown). However, we observed that three proteins, LegC2/YlfB-GFP, LegC7/YlfA-GFP and LegC3-GFP exhibited specific localization patterns. As shown in Figure 4A, LegC3-GFP is found associated with the yeast plasma membrane as well as punctate vacuolar structures within the cell. Compared to cells expressing GFP alone, LegC3-GFP expression also caused a partial fragmentation and tubulation of the vacuole membrane as seen by the FM4-64 staining pattern. The punctate LegC3-GFP containing structures also co-localized with parts of the fragmented vacuole. In contrast, LegC2/YlfB-GFP and LegC7/YlfA-GFP formed nodular structures on the vacuole and co-localized heavily with the FM4-64 stain. The accumulation of FM4-64 in the pre-vacuole is similar to what is observed in Class E VPS mutants, indicating that these proteins block endosome maturation and trafficking to the yeast vacuole via the multivesicular body (MVB) pathway. Western blots on whole cell lysates using a polyclonal rabbit anti-GFP antibody showed stable expression of all three proteins, although LegC7/YlfA is expressed to a lower level than LegC3 and LegC2/YlfB (Figure 4B).

To determine if the localization of the YlfA/LegC7 and LegC3 proteins depends on other components of the Vps/ESCRT complex, we expressed these constructs in a collection of 25 VPS mutants containing null mutations in genes that encode components of the Vps/ESCRT complexes; (Δ*vps1,* Δ*vps2,* Δ*vps4,* Δ*vps16,* Δ*vps17,* Δ*vps18,* Δ*vps20,* Δ*vps22,* Δ*vps23,* Δ*vps24,* Δ*vps25,* Δ*vps27,* Δ*vps28,* Δ*vps35,* Δ*vps36,* Δ*vps37,* Δ*vps44,* Δ*vps46,* Δ*vps60,* Δ*bro1,* Δ*doa4,* Δ*hse1,* Δ*sec28,* Δ*snf7,* and Δ*vta1*). We observed that in some cases YlfA/LegC7 and LegC3 exhibited more intense vacuolar staining, but not mis-localization (data not shown). This result indicates that these proteins do not require other known scaffolding components or docking proteins of the ESCRT complex for their association with the yeast vacuole and are likely tethered to endosomes via putative transmembrane domains, and not through interactions with components of the multivesicular body pathway.

### The Hydrophobic Domains of LegC2/YlfB, LegC7/YlfA and LegC3 are required for Protein Localization and Modification of Vacuolar Morphology

**Figure 5:**
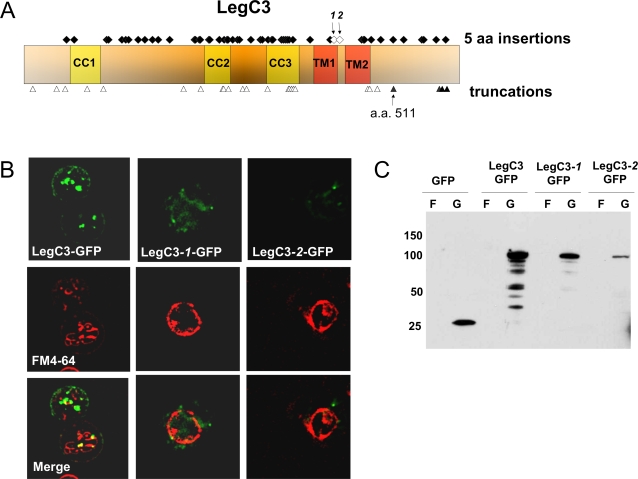
A Hydrophobic Region at the C-terminus of LegC3 is Essential for its Localization in Yeast. (A) LegC3 was mutagenized using the GPS-LS Linker Scanning kit (New England Biolabs) to generate a library of 60 distinct mutant proteins with five amino acid insertions and 27 distinct truncation products. The positions of the 60 insertions are indicated by diamonds and the 27 truncations by triangles together with the LegC3 structural predictions. The open diamonds and triangles represent insertion and truncation sites, respectively, that exhibited an altered LegC3-GFP localization. (B) Epifluorescence microscopy showing specific localization patterns for LegC3-1 and LegC3-2 compared to wild-type LegC3. (C) Whole cell lysate immunoblot using a rabbit polyclonal antibody against GFP showing that the LegC3-1 and LegC3-2 mutant proteins are stably expressed under galactose induction.

In order to determine which regions of LegC3 are required for producing a VPS defect and localization in yeast, we analyzed a library of LegC3 mutations. Using a transposon-based delivery system, we obtained 60 insertions of 15 base pairs each: 33 mutations were found to contain in-frame codon insertions and 27 contained out-of-frame (nonsense) insertions (Figure 5A). We screened the resulting yeast library for LegC3-GFP localization and vacuolar morphology (as determined by FM4-64 staining). As shown in Figure 5B, we found that two insertion mutants at amino acid positions 390 (LegC3-1-GFP) and 395 (LegC3-2-GFP) altered the protein localization and vacuolar morphology. These two mutant proteins had transposon insertions within the hydrophobic domain, exhibited a diffuse GFP signal, and failed to localize to the plasma membrane. Additionally, the vacuole of cells expressing these mutant versions of LegC3-GFP appeared to be more similar to that of strains that express GFP alone. Western blots were performed on lysates of yeast cells grown in fructose and galactose to confirm that the mutant LegC3-GFP proteins were present at the predicted sizes. As shown in Figure 5C, full length LegC3-1-GFP and LegC3-2-GFP were expressed following galactose induction, although the level of each protein was slightly reduced compared to LegC3-GFP. In addition to the hydrophobic domain, we also observed that the first 511 amino acids of the protein (out of 558) are sufficient for causing the VPS defect, as the first permissive truncation that still caused CPY-Invertase mis-secretion was at this position (data not shown).

**Figure 6:**
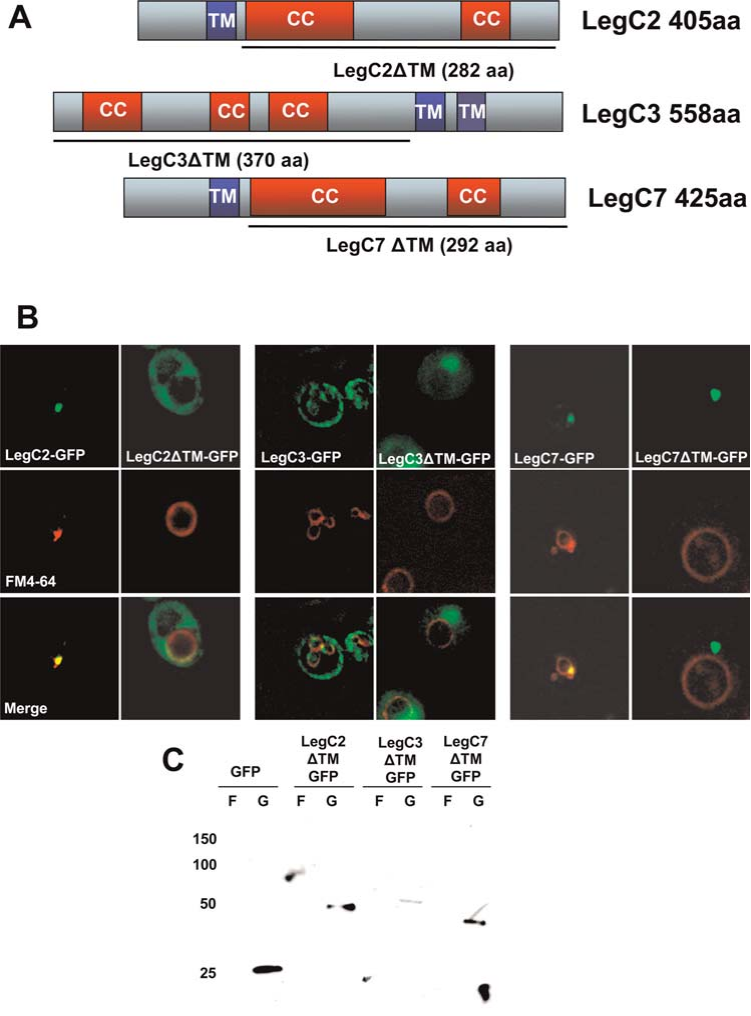
The Predicted Transmembrane Domains of LegC2, LegC3, and LegC7 are Required for Their Localization in Yeast. (A) Constructs of LegC2, LegC3 and LegC7 were designed to exclude the predicted transmembrane regions. The solid line drawn beneath each gene represents the amino acid sequence included in each final construct. The truncated constructs were translationally fused to *gfp* and transformed into NSY01 for expression. (B) Epifluorescence microscopy showing localization patterns. (C) Whole cell lysate immunoblot using a rabbit polyclonal antibody against GFP showing the truncated proteins.

Because the amino acid insertions in LegC3-GFP that altered the protein localization were within a hydrophobic region predicted to be a an α-helical transmembrane domain, we decided to test if the hydrophobic regions of LegC2/YlfB and LegC7/YlfA were required for protein localization. As shown in Figure 6A and 6B, deleting the hydrophobic domain of LegC2/YlfB-GFP caused the protein to become cytosolic. LegC7/YlfAΔTM-GFP, in contrast, formed a single bright punctate structure distinct from its original localization pattern on the vacuolar membrane. Furthermore, this protein did not co-localize with the vacuole membrane, as was the case with full-length LegC7/YlfA-GFP. As predicted based on the transposon-mutant insertion data for LegC3-GFP, genetic deletion of the two predicted transmembrane domains of this protein resulted in cytosolic localization and it no longer caused structural changes in the yeast vacuole membrane. Western immunoblots confirmed that the deletion mutants fused to GFP were expressed as full-length proteins of the predicted molecular weight (Figure 6C). These results demonstrate that the hydrophobic regions of these Leg proteins are required for their proper localization in yeast, perhaps by anchoring them to endocytic vesicles.

### LegC3, LegC2/YlfB, and LegC7/YlfA localization in mammalian cells

**Figure 7:**
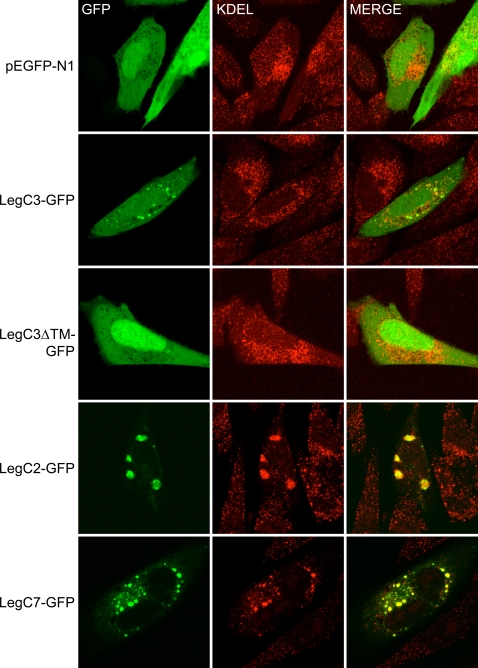
Localization of Leg-GFP Fusion Proteins and KDEL-containing proteins in transiently transfected CHO-FcγRII cells. CHO-FcγRII cells were transfected with plasmids expressing GFP (pEGFP-N1), LegC3-GFP (pRG6), LegC3ΔTM (pRG7), LegC2-GFP (pRG8) or LegC7 (pRG10), as indicated on the left of each row. Representative confocal images were acquired demonstrating the location of GFP and proteins containing KDEL motifs as determined by immuno-fluorescent staining. Merged images are shown in the right column where yellow color designates overlap of the green and red channels.

The LCV is known to recruit vesicles derived from the ER (endoplasmic reticulum) and Golgi complex while excluding markers of early endosomes and lysosomes [21],[64]. The results we obtained in the yeast assays are consistent with LegC3, LegC2/YlfB, and LegC7/YlfA localizing to trafficking organelles and possibly playing a direct role in perturbing normal vacuolar maturation. In order to determine if similar phenotypes are observed in higher eukaryotes, each gene was transiently expressed in mammalian CHO cells [65] as a C-terminal GFP fusion protein. Unfused GFP was found throughout the cytoplasm. In contrast, LegC3-GFP formed multiple punctate structures, although some degree of cytoplasmic expression was also evident (Figure 7). Concordant with the results obtained in yeast cells, the formation of punctate structures was dependent upon the hydrophobic domain of LegC3, as deletion of this domain resulted in a diffuse cytoplasmic distribution. To determine if LegC3-GFP expression was associated with a specific compartment of the endocytic pathway, immuno-fluorescent staining was performed using antibodies against ER (α-KDEL) and lysosomal (α-LAMP-1) markers. As shown in Figure 7, the LegC3-GFP structures had no obvious co-localization with KDEL-containing proteins and excluded the lysosome-associated marker LAMP-1.

**Figure 8:**
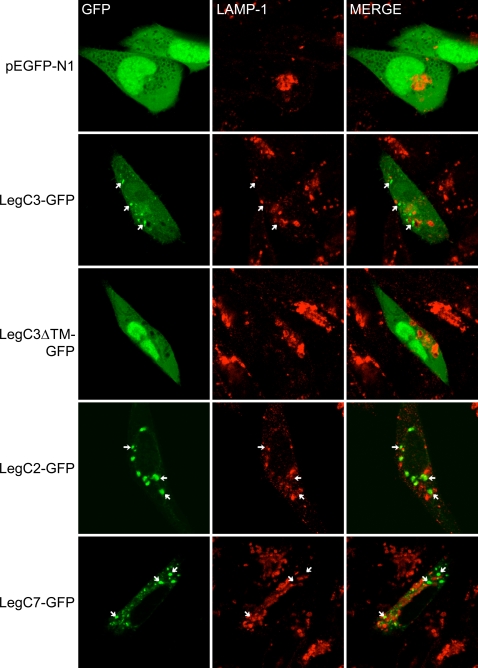
Localization of Leg-GFP Fusion Proteins and LAMP-1 in transiently transfected CHO-FcγRII cells. CHO-FcγRII cells were transfected as in Figure 7. Representative confocal images demonstrating the location of GFP and LAMP-1 staining are shown. When present, arrows indicate the location of GFP-positive structures to distinguish these from proximal LAMP-1 lysosomes. Merged images are shown in the right column. The lack of yellow color in merged verifies a lack of colocalization between GFP-positive structures and lysosomes.

In contrast to LegC3-GFP, LegC2/YlfB-GFP expression resulted in the formation of large vacuole-like compartments. Interestingly, these vacuole-like compartments resembled the LCV in their strong co-localization with KDEL-containing proteins and exclusion of LAMP-1 (Figure 8). Similarly, expression of LegC7/YlfA-GFP localized within vacuole-like compartments, although the relative size of these compartments was smaller than that seen with LegC2/YlfB-GFP. Consistent with the idea that LegC2 and LegC7 may have similar functions, the LegC7/YlfA-GFP-containing compartments also co-localized with KDEL proteins and did not co-localize with LAMP-1.

### Accumulation of Vesicles with Undigested Material in *D. discoideum* Expressing GFP-LegC3-His7X

**Figure 9:**
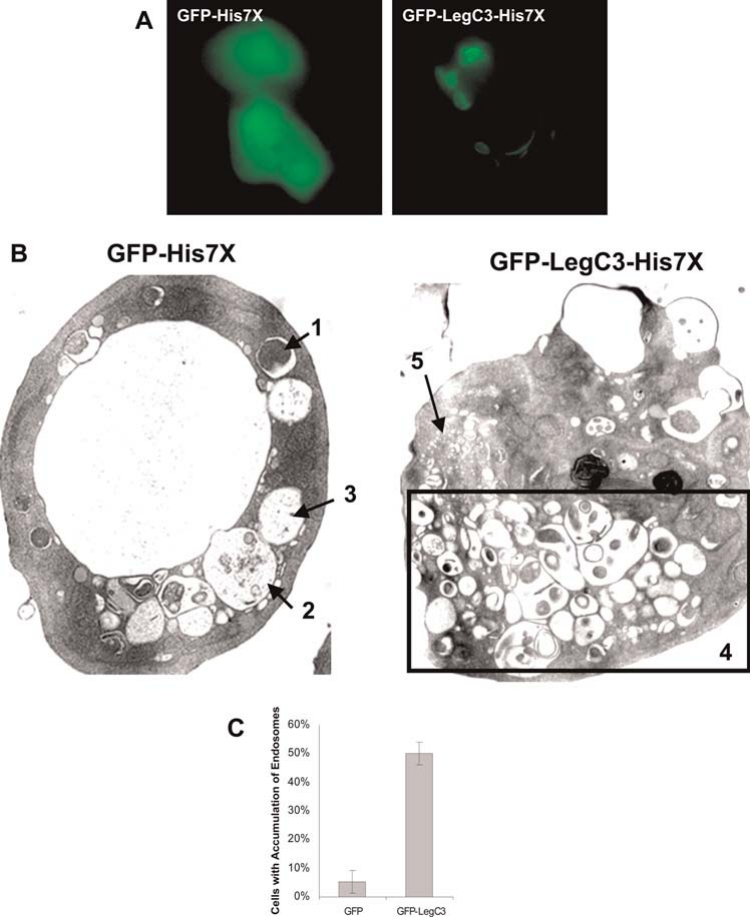
Expression of GFP-LegC3-His7X and GFP-His7X in *D. discoideum.* (A) *D. discoideum* cells containing *GFP-His7x*, or *GFP-LegC3-His7x* under the control of a Tc-repressible promoter were grown with the presence of 10 µg/mL of Tc for 3 days at 25°C. The cells were washed in SorC buffer and incubated for an additional 8–10 hours in media without tetracycline. Expression of the fusion proteins was visualized under epifluorescence microscopy. (B) *D. discoideum* cells expressing hybrid fusion proteins were sorted for GFP+ cells after 10 hours of induction. The samples were then fixed and processed for electron microscopy using osmium tetroxide staining. Cells expressing GFP-His7X contain a mixture of vesicles with (1) undigested material, (2) partly digested material and (3) completely digested material. At least half of the cells expressing GFP-LegC3-His7X contain (4) an abnormally high number of vesicles with partly digested contents. What appears to be unfused pro-lysosomes (5) are segregated to a separate portion of the cell. (C) At least 100 cells of each sample were analyzed for abnormal fine structures. The percentage of GFP-LegC3-His7X expressing cells containing abnormal vesicle accumulation versus GFP-His7X expressing cells. 95% confidence intervals are indicated above.

Due to the dramatic and novel effects of LegC3-GFP expression on the yeast vacuole, we wanted to determine the effect of LegC3 expression in a protozoan host of *L. pneumophila*, *D. discoideum*. We took advantage of both epifluorescence microscopy and transmission electron microscopy (TEM) to examine cells expressing GFP-LegC3-His7X and GFP-His7X, each under the control of a tetracycline-regulated promoter. When examined by epifluorescence microscopy, cells expressing GFP-LegC3-His7X exhibited both vesicular and lamellar staining patterns, whereas a diffuse cytosolic staining pattern was seen with the GFP-His7X control (Figure 9A). We next isolated a population of cells that were GFP positive using a fluorescence activated cell sorter and processed them for TEM using standard techniques for osmium tetroxide staining. As shown in Figure 9B, cells expressing GFP-His7X alone had normal fine structures and vesicles. Endosomes containing undigested (labeled 1), partially digested (labeled 2) and fully digested material (labeled 3) are visible. In contrast, cells expressing GFP-LegC3-His7X presented an abnormally large number of vesicles with undigested material (labeled 4). Furthermore, structures that appeared to be un-fused pro-lysosomes can also be seen in these cells (labeled 5). This phenotype was observed in at least 50% of the 100 cells analyzed and in less than 4% of the cells expressing GFP-His7X alone (Figure 9C). These results suggest that expression of GFP-LegC3-His7X may affect the steady state formation and degradation of endosomal contents in *D. discoideum*.

## Discussion

Professional phagocytes have developed pathways dedicated to killing internalized microorganisms by rapidly fusing phagosomes with acidic and hydrolytic vesicles known as lysosomes. While the fate of most bacteria that encounter phagocytic cells is death and degradation, some have evolved mechanisms to avoid this pathway. Many pathogens simply prevent phagocytosis, some puncture the phagosome and escape into the cytosol, yet others, such as *L. pneumophila,* survive by blocking phagosome maturation altogether. However, the formation of a replication-competent compartment is quite involved; *L. pneumophila* modifies its phagosome in many different ways to transform it into a nutrient-sufficient, non-acidic and non-hydrolytic environment. This process is believed to require the delivery of specific effector proteins into host cells. It is therefore important to identify and characterize the mechanism of action of these effectors to understand how *Legionella pneumophila* replicates inside host cells. Previously, we reported the identification of 45 genes in *L. pneumophila* predicted to encode proteins with distinct eukaryotic motifs (*leg* genes) that may have been acquired via interdomain horizontal gene transfer [43].

In this study, we set out to determine which of the *leg g*enes actually encode translocated effector proteins. We found that 25 Leg proteins are substrates of the *L. pneumophila* Icm-Dot translocation system, in addition to 8 previously identified Leg substrates. While several *L. pneumophila* effector proteins have been found to interact with the early secretory machinery (such as RalF, LidA and DrrA/SidM), there is considerable lack of information on what effector proteins may promote a block in phagosome maturation. Here we show that three effector proteins, LegC2/YlfB, LegC7/YlfA and LegC3, may contribute to this process. When expressed in yeast, these predicted transmembrane proteins seem to block the pre-vacuole compartment, preventing endosomes from maturing and fusing to the vacuole. This can be seen by the accumulation of the styryl dye FM4-64 in a compartment that contains these effector proteins and that are associated with the vacuole. A block in endocytosis is further supported by the observation that LegC7/YlfA and LegC3 cause mis-sorting of CPY-Invertase, a protein that normally trafficks to the vacuole. Interestingly, LegC2/YlfB, a paralog of LegC7/YlfA, did not cause a detectable CPY-Invertase defect despite its vacuole-associated localization within cells. It is possible that LegC2/YlfB is located within membranous structures but lacks an appropriate binding partner required for disrupting vacuolar maturation. Moreover, CPY is only one of many cargos that traffic to the vacuole, and certain cargos are delivered with varying genetic requirements [66]. It will be interesting to determine the exact nature of the effect of these proteins in the endocytic network and if they perform similar roles during infection of natural host cells.

Although we did not observe a growth defect in cells that express either LegC7/YlfA or LegC2/YlfB, these genes were previously identified based their lethality in a similar yeast-based assay [36]. This discrepancy may be explained by the different vectors utilized; the previously utilized vector, pJG4-5, is a high-copy plasmid that creates N-terminal fusions to a nuclear localization signal and a B42 transcriptional activation domain, which may cause effector proteins to be expressed at different levels and localize to different organelles. The level of heterologous gene expression can thus have important consequences on the effects of recombinant *L. pneumophila* proteins in yeast-based screens. Our finding that LegC5, LegC8 and LepB caused growth defects in yeast may thus still prove to be interesting and useful in finding out which cellular pathways they affect. Mutations in some VPS genes, for instance, cause varying ranges of growth defects in yeast (personal communications, Scott Emr). It is intriguing that LepB, a protein that may have a role in the release of the bacteria from protozoan (but not mammalian) hosts, strongly inhibits growth in yeast. LepB has recently been shown to also act as a GTPase-activating protein functioning to remove Rab proteins from the LCV [27]. The lethal effect of LepB expression may open the way for the study of the effects of this effector protein on exocytosis pathways in yeast.

One caveat to yeast-based screens is that overexpressed proteins, particularly those carrying predicted transmembrane or hydrophobic domains, may produce artificial results. However, a recent study has shown that the overproduction of integral-membrane proteins in yeast seems to cause effects that are mainly protein-specific [67]. In addition, we only observed phenotypes for a small number of effector proteins, even though many contained hydrophobic domains. One notable conclusion from our studies is that only effector proteins that contain coiled-coil domains presented phenotypes in the assays we utilized. This protein-binding domain is often found in eukaryotic trafficking proteins and in many VPS proteins in yeast. This raises the possibility that effector proteins with other domains either affect pathways we did not test, or do not function in yeast.

TEM analysis of *D. discoideum* cells expressing GFP-LegC3-His7X revealed fine structure modifications that are difficult to observe using other types of microscopy. Here we show that expressing this protein in *D. discoideum* causes the accumulation of many vesicles with undigested matter. This is of direct relevance to *L. pneumophila*'s lifestyle, since this organism is known to alter membrane trafficking in such a way that endocytic maturation is locally halted. One complicating factor, nonetheless, is the finding that a certain portion of the overexpressed LegC3 seems to remain localized to lamellar structures that may be ER. Others have demonstrated that the overexpression of certain proteins in *D. discoideum* may lead to the accumulation of aggregates in the ER cisternae [68]. However, the TEM pictures of these aggregated ER cisternae are quite distinct from the TEM images of cells expressing GFP-LegC3-His7X. Furthermore, the accumulation of proteins in the ER does not interfere with endocytic vesicles in the previous studies. The dramatic effects that LegC3 causes in both yeast vacuolar membranes and *D. discoideum* endosomes suggest that this effector plays an important role in *L. pneumophila's* ability to arrest phago-lysosome fusion.

Following ectopic expression of LegC3, LegC2/YlfB, and LegC7/YlfA as GFP fusion proteins in mammalian CHO cells, specific intracellular localization patterns were observed. LegC3-GFP was concentrated within multiple distinct, punctate structures and this localization was dependent on the presence of the hydrophobic, putative transmembrane domain. The LegC3-containing structures did not appear to co-localize with either the ER or early secretory vesicles (based on α-KDEL staining) or late endosomes/lysosomes. As such, we cannot currently determine whether LegC3 is specifically targeted to a specific organelle or pathway in mammalian cells. It is interesting to note, however, that the punctate localization of LegC3 within mammalian cells is similar to that observed in the yeast screen, suggesting that LegC3 association with membranes plays an important role in its function in each cell type.

In contrast to what was observed with LegC3-GFP, ectopic expression of LegC2/YlfB-GFP and LegC7/YlfA-GFP in mammalian cells induced the formation of and appeared within large vesicle-like structures. In each case, these structures co-localized with KDEL-containing proteins and excluded the lysosomal marker LAMP-1, concurring with previous results reported for LegC7/YlfA [36]. Notably, these vacuole-like structures are similar to the LCV in their association with ER-resident proteins and exclusion of LAMP-1, suggesting that each protein is sufficient to remodel vacuolar trafficking, perhaps to benefit the intracellular survival of *L. pneumophila*.

Drawing conclusions about individual effector proteins based on their ectopic expression in the absence of the infectious organism may be regarded with skepticism. However, we believe this is a useful strategy to assist in determining the function and significance of effector proteins in the infectious process. Single or even multiple deletions of putative effector proteins rarely have any observed effect on the ability of *L. pneumophila* to infect and replicate in host cells. In fact, although our results might suggest a significant role for LegC2/YlfB and LegC7/YlfA in modulating the LCV, strains deleted for both genes replicated in protozoan and mammalian host cells with similar kinetics to wild type [36]. The lack of an intracellular growth phenotype in single or double knockout strains is presumed to be due to a redundancy within the pool of translocated effectors, where multiple proteins may target the same pathway(s). The extensive set of homologous effectors present in *L. pneumophila* may be explained by their origin. If effectors were originally acquired via horizontal gene transfer and then duplicated over time, this set of “foreign” genes could serve as a pool for the selection of effectors whose functions are fine-tuned for specific interactions and/or specific host species. What may first look like “redundancy” may in fact be a source of untapped resources that allows this pathogen to evolve and survive in several distinct niches. The genome sequence of *L. pneumophila* may thus represent just a snap-shot of effector genes during the process of evolution.

At present, it is difficult to determine which host pathways are appropriated by specific effector proteins. Nevertheless, the importance of the Icm-Dot TFSS for the virulence of *L. pneumophila* strongly supports the belief that effector proteins translocated into host cells are required to prevent normal bactericidal responses. Our results demonstrate that LegC3, LegC2/YlfB, and LegC7/YlfA can affect normal vacuolar trafficking in yeast, protozoan, and mammalian cells. The data presented here suggest that endosomal trafficking and particularly the Vps/ESCRT pathway may play a role in the construction or maintenance of the Legionella-containing vacuole. Additional work should provide information about the involvement of this or other conserved host cell pathways in the intracellular lifestyle of *L. pneumophila*.

## Materials and Methods

### Bacterial and Yeast Strains

The bacterial and yeast strains used in this study are listed in Table 2. With the exception of KS79, bacterial strains, media and antibiotics are described elsewhere [43]. KS79 is an isogenic Δ*comR* derivative of JR32. This strain was generated by the isolation of a Tn903dII*lacZ* insertion in the *comR* gene followed by the removal of the entire ORF, including the transposon, to generate an unmarked *comR* mutant. Generation of the yeast strain NSY01 is described elsewhere [38].

**Table 2:** Strains Used in This Study

Strains	Relevant Genotypes	Reference
JR32	Philadelphia-1, Sm^R^, r-, m+	[73]
KS79	JR32 *ΔcomR*	This study
NSY01	BHY10 diploid	[38]
*D. discoideum AX2*	Standard Axenic Strain	[74]

### Construction of Plasmids

Descriptions of all cloning strategies are described below, and a complete list of plasmids used in this study is provided in Table 1.

The *blaM* gene encoding the mature form of TEM-1 beta-lactamase (residues 24–286) was amplified by PCR from pUC18 with forward and reverse primers introducing a ribosome-binding site and a KpnI-SmaI-BamHI-XbaI polylinker at the 3′ end. The PCR product was cloned into EcoRI/HindIII digested pMMB207C [33] to generate plasmid pXDC61. The *leg* genes were PCR-amplified and cloned in frame with the beta-lactamase at the KpnI-XbaI sites. The *leg* genes that contain one of these restriction sites were cloned into 76XbaI (legA10-S2-T), KpnI (legC7-C8-U1) or BamHI site (LegK1). The resulting plasmids were then introduced by natural transformation into KS79 (JR32 *ΔcomR*), KS79 *dotA::Tn*903dII*lacZ* or KS79 *icmT::Tn*903dII*lacZ*.

The low-copy, galactose inducible plasmid pBM272 is described elsewhere [60]. The *gfp* S65T variant was amplified by PCR with primers containing the restrictions sites HindIII/SalI. The PCR product was double-digested with these enzymes and cloned into the HindIII/SalI sites of pBM272 to generate pKS84. 30 *leg* genes and LepA and LepB were amplified by PCR with primers containing the appropriate restriction sites and inserted either into the BamHI site or HindIII site of pKS84 to generate *leg-*GFP fusions. To create un-fused version of these proteins, *legC2, legC3, legC5, legC7, lepA* and *lepB* were also cloned into pBM272 using identical restriction sites. These plasmids were introduced into NSY01 by the Lithium Acetate transformation method [69].

pKS138 was constructed by inserting a DNA fragment encoding a His7x tag into the Sph1 and Mlu1 sites of pMB38-GFP (a generous gift from Barbara Weissenmayer at the Berlin University). *legC3* was amplified by PCR with primers containing BamHI restriction sites subsequently digested with the appropriate enzymes. The product was inserted into the BamHI site of pKS138. Plasmids pKS138 and pKS140 were transformed into *D. discoideum* AX2 MB35 by electroporation.

For eukaryotic expression experiments, *legC3, legC3ΔTM, legC2*, and *legC7* were amplified by PCR using oligonucleotides that introduced an NheI site and a Kozak consensus sequence (gccgccaccATGgtg) immediately before each gene at the 5′ end and a KpnI site at the 3′. PCR products were sub-cloned into pEGFP-N1 (Clontech) at the NheI and KpnI sites using unidirectional cloning to generate translational Leg-GFP fusion proteins under the transcriptional control of the CMV I/E promoter.

### TEM Translocation assays and Immunoblots

J774 cells grown in RPMI containing 10% fetal calf serum (FCS) were seeded in black clear-bottom 96-well plate at 1x10^6^ cells/well 24 hours prior to infection. *L. pneumophila* strains carrying the various *blaM* fusions were grown on CYE plates containing choramphenicol and single colonies were then streaked on CYE plates containing chloramphenicol and 0.5 mM Isopropyl β-D-1-thiogalactopyranoside (IPTG) and grown for 24 hours to induce expression of the hydrid proteins. 10 µL of bacteria re-suspended in RPMI at 5×10^8^ cells/mL were used to infect J774 cells (MOI = 50). After centrifugation (600g, 10 minutes) to initiate bacterial-cell contact the plate was shifted to 37°C and incubated for one hour with CO_2_ exchange. Cell monolayers were loaded with the fluorescent substrates by adding 20 µl of 6x CCF4/AM solution (LiveBLAzer-FRET B/G Loading Kit, Invitrogen) containing 15 mM Probenecid (Sigma). The cells were incubated for an additional 2 hours at room temperature. Fluorescence was quantified on a Victor microplate reader (Perkin-Elmer) with excitation at 405 nm (10-nm band-pass), and emission was detected via 460-nm (40-nm band-pass, blue fluorescence) and 530-nm (30-nm band-pass, green fluorescence) filters. Translocation was expressed as the emission ratio at 460/530 nm to normalize the ß-lactamase activity to cell loading and the number of cells present in each well. The presented data are mean values of the results from triplicate wells from two to three experiments. The cells were also visualized by fluorescence microscopy using an inverted microscope equipped with the Beta-lactamase ratiometric filter set (Chroma).

### Yeast Microscopy and FM4-64 Staining

*S. cerevisiae* cells expressing effector proteins were grown overnight in 3 mL cultures at 30°C in SC-Ura/fructose. The cultures were then back-diluted to A_600_ of 0.4–0.6 in 3 mL of SC-Ura/galactose and grown to A_600_ of 0.8–1.2. For vacuolar staining, the induced cultures were centrifuged and re-suspended in YP medium containing 2% galactose. N-(3-triethylammoniumpropyl)-4-(p-diethylaminophenylhexatrienyl)-pyridinium dibromide, FM4-64 (Molecular Probes), was added to a final concentration of 40 µM. After 15 minutes of incubation at 30°C, the cells were washed twice and re-suspended in 3 mL of fresh YP medium containing 2% galactose. We allowed cells to incubate for an additional 45–60 minutes before visualization. We visualized cells under a Nikon Eclipse TE200 at 100x using an oil immersion phase-contrast objective. For fluorescence microscopy, we utilized filter sets for fluorescein isothiocyanate and Texas red. For Z-stack image acquisition, we used a Hamamatsu digital camera with a computer-controlled Z-axis drive. We acquired approximately 20 Z-sections every 0.3–0.4 µm (spanning 6–8 µm) and performed volume deconvolution using Improvision's Open Lab software.

### Invertase Assays

The quantitative and qualitative invertase assays were based on a previous published methodology [59]. For the qualitative assay, cells streaked on SC-Ura/galactose or SC-Ura/fructose plates were incubated for 4 days at 30°C. The plates were overlaid with 0.75% agar solution containing 125 mM sucrose, 100 mM sodium acetate buffer (pH 5.5), 0.5 mM *N-*ethylmaleimide (NEM), 10 µg/mL horseradish peroxidase, 8 units/mL glucose oxidase, and 2 mM *O*-dianisidine. After 5–15 minutes, pictures were taken with a Sony Cybershot camera. Image contrast was adjusted using the open source software GIMP. For the quantitative assays, cells were grown in liquid SC-Ura/galactose to stationary phase and split into two samples for measurement of total invertase activity and exogenous invertase activity. The assay was scaled down and performed in 96-well plates. To measure total invertase activity, one of the samples was first lysed by the addition of Triton X to a final concentration of 2% followed by 4 cycles of freeze-thaw. 20 µL of each sample was then mixed with 20 µL of a 0.1 M Sodium Acetate (pH 4.9) solution and 5 µL of a 0.5 M sucrose solution followed by incubation at 30°C for 30 minutes. To inactivate the invertase enzyme, 30 µL of 0.2 M K_2_HPO_4_ (pH 10.0) was added followed by heating to 95°C for 10 minutes. The samples were then allowed to cool down to room temperature and mixed with 150 µL of the glucostat reagent [59]. The reactions were stopped by the addition of 200 µL of 6M HCl. The A_540nm_ of all samples was measured using a microplate reader (Molecular Device). The units of invertase enzyme were calculated using a standard glucose curve and the following definition: one unit of invertase is the amount of enzyme that hydrolyzes sucrose to produce 1 µM of glucose per minute at 30°C.

### Generation of LegC3 Insertion and Truncation Library

The LegC3 mutant library was generated using the GPS-LS Linker Scanning kit (New England Biolabs). We followed the manufacturer's protocol to generate a collection of 92 transposon-generated mutations in the LegC3 gene. On average, 2/3 of the mutants generated have 15 bp insertions in the gene of interest while 1/3 of them create truncated products. We sequenced the whole library (Genewiz, inc.) to determine the exact nature and location of each mutation. The 92 plasmids were then transformed into NSY01 for further analysis. The LegC3-1-GFP mutant protein has an insertion at amino acid position 390 of VFKQS and LegC3-2 an insertion at amino acid position 395 of CLNTF.

### Yeast Immunoblots

Yeast strains were grown on SC Ura/Fructose plates for two days at 30°C. Several colonies were picked and spread on SC Ura/Galactose plates and incubated for three days at 30°C. Yeast cells were scraped with 10 mL of 50 mM Tris-HCl pH 7.5 and then centrifuged at 10,000 rpm for 5 minutes. The cells were then re-suspended in 2 mL of 50 mM Tris-HCl pH 7.5, divided into 0.5 mL aliquots and frozen at −80°C. The frozen pellet was then re-suspended in an additional 0.5 mL of 50 mM Tris-HCl pH 7.5 and the absorbance at 600 nm measured. An amount equivalent to OD 5 in a total volume of 300 µL of Tris-SB was boiled for 5 minutes. The sample LegC7-GFP was concentrated ten times relative to this measurement. 15 µL was loaded onto 4–15% polyacrylamide gradient gels. Following electrophoeresis, the proteins were transferred to a nitrocellulose membrane and processed for immunoblots using an affinity-purified rabbit polyclonal antibody to GFP at a 1:2,000 dilution [70].

### *D. discoideum* Epifluorescence Microscopy

*D. discoideum* cells containing pKS138 and pKS140 were grown for three days with slow shaking in a 22°C water bath. The cells were then washed 3 times with an equal volume of SorC buffer. The cells were then re-suspended in HL5 medium and allowed to grow for an additional 6–10 hours at 22°C [71]. We visualized cells under a Nikon Eclipse TE200 at 100× using an oil immersion phase-contrast objective. For fluorescence microscopy, we utilized filter sets for fluorescein isothiocyanate.

### *Dictyostelium* Electron Microscopy

*D. discoideum* cells containing pKS138 and pKS140, grown for three days with slow shaking in a 22°C water bath, were washed 3 times with an equal volume of SorC buffer, resuspended in HL5 medium and grown for an additional 10 hours at 22°C. Cells were spun down at low speed, re-suspended in SorC buffer, prepared for sorting at the Columbia University Cell Sorting facility, and one million GFP^+^ cells of each strain were sorted. The cells were plunged into ice and prepared for TEM according to the O R Anderson method [72]. An equal volume of suspended cells was added to a 6% TEM grade glutaraldehyde [Ladd 20215] solution containing HL5 and SorC buffer, gently placed on ice for 30 minutes, resedimented by gentle centrifugation, and the pellet was post-fixed with 1 mL of 2% osmium tetroxide solution [Ladd 55090] in cacodylate buffer. The post-fixed cells were sedimented by gentle centrifugation. The pellet was gently resuspended in 0.8% ionagar sol at 42°C by flicking the tube, rapidly pelleted in the agar sol by centrifugation and the enrobed pellet solidified by placing the tube in an ice bath. The enrobed pellet was removed from the tube with a spatula, washed in water, and dissected into 1 mm^3^ segments. The segments were washed in water, dehydrated in a graded aqueous acetone series, infiltrated with and embedded in low viscosity epon, placed in BEEM capsules containing the epon, and polymerized for 24 hours at 72°C. Ultrathin sections, obtained with a Porter-Blum MT-2 Ultramicrotome fitted with a diamond knife, were collected on uncoated copper grids, post-stained with Reynold's lead citrate, and observed at 60 kV with a Philips 201 TEM.

### Mammalian immuno-staining and confocal microscopy

CHO-FcγRII cells [65] were maintained in α-MEM media containing 10% FBS. Cells were seeded at 2×10^4^ cells/well on 12 mm coverslips and incubated overnight. The following day, cells were transfected with plasmid DNA using Fugene HD (Roche) according to the manufacturers instructions. 16 hours later, cells were fixed with 3.7% PBS-buffered formalin for 20 minutes, washed with PBS, and blocked/permeabilized with 2% BSA in PBS containing 0.1% saponin. All subsequent buffers, including wash buffers, contained 0.1% saponin. Cells were incubated with blocking buffer containing primary antibodies against KDEL (1:200) (Santa Cruz Biotechnology) or UH1, a monoclonal antibody against hamster Lgp-A/LAMP-1 (1:200) (DSHB, University of Iowa) for 1 hour, washed, and incubated with goat anti-mouse Alexa Fluor 564 (1:500) (Invitrogen) for 30 minutes. After washing, coverslips were fixed to glass slides using Vectashield HardSet mounting media (Vector Labs) for confocal microscopy. Confocal images were acquired with a Zeiss LSM510 Meta laser scanning microscope. Captured images were processed and merged using the open-source NIH software, ImageJ (http://rsb.info.nih.gov/ij/index.html).

## Supporting Information

Figure S1 Detection of Leg-GFP proteins in yeast by immunoblot analysis. (A) Table A shows the plasmids that did not result in detectable fluorescence in yeast after galactose induction as determined by fluorescent microscopy. (B) Table B shows plasmids that did not produce a detectable band after immunoblotting but did show low levels or diffuse GFP localization by fluorescent microscopy. (C, D, E, F) Immunoblots on yeast lysates following galactose induction. The expression construct and predicted molecular weight are shown above each respective lane. Immunoblots were performed as described in Materials and Methods.
(2.72 MB TIF)
