# Correction: *Legionella* Eukaryotic-Like Type IV Substrates Interfere with Organelle Trafficking

**DOI:** 10.1371/annotation/f079872b-7bce-41fe-bc16-c2e45d44b19e

**Published:** 2010-03-24

**Authors:** Karim Suwwan de Felipe, Robert T. Glover, Xavier Charpentier, O. Roger Anderson, Moraima Reyes, Christopher D. Pericone, Howard A. Shuman

The caption for Figure 3 was incorrect. The correct caption for Figure 3 is:

Yeast cells containing expression vectors for LegC5-GFP, LegC8-GFP, LepB-GFP fusion proteins or GFP alone were spotted in 10-fold serial dilutions on SC-ura/fructose or SC-ura/galactose plates. Under conditions that induce expression of the hybrid protein (galactose), LegC5-GFP, LegC8-GFP and LepB-GFP impair yeast growth. Under non-inducing conditions (fructose), all strains grow comparably well.

