# Correction: *Legionella* Eukaryotic-Like Type IV Substrates Interfere with Organelle Trafficking

**DOI:** 10.1371/annotation/c7438c1b-5b65-4f4c-aaa3-062909e89525

**Published:** 2010-03-24

**Authors:** Karim Suwwan de Felipe, Robert T. Glover, Xavier Charpentier, O. Roger Anderson, Moraima Reyes, Christopher D. Pericone, Howard A. Shuman

The caption for Figure 2 was incorrect. The correct caption for Figure 2 is:

(A) Yeast strains expressing CPY-Inv and LegC3-GFP, LegC7-GFP, GFP or VPS4E233Q were streaked on SC-ura/fructose or SC-ura/galactose and grown for four days at 30°C. As described in the Materials and Methods, an agar solution containing chromogenic reagents to detect invertase activity was overlaid on the plates. The secretion of the CPY-Invertase hybrid is detected by the formation of a brown precipitate. (B) Quantitative assay for invertase secretion. Liquid cultures were grown to stationary phase and secreted versus total invertase activity was measured as described in Material and Methods. The results shown are of a representative experiment done in triplicate +/-; standard deviations.

