# Supplementary figures and images for: Legionella Eukaryotic-Like Type IV Substrates Interfere with Organelle Trafficking
Source: PLoS Pathog. 2008 Aug 1;4(8):e1000117. doi: 10.1371/journal.ppat.1000117 (PMC2475511; doi:10.1371/journal.ppat.1000117)

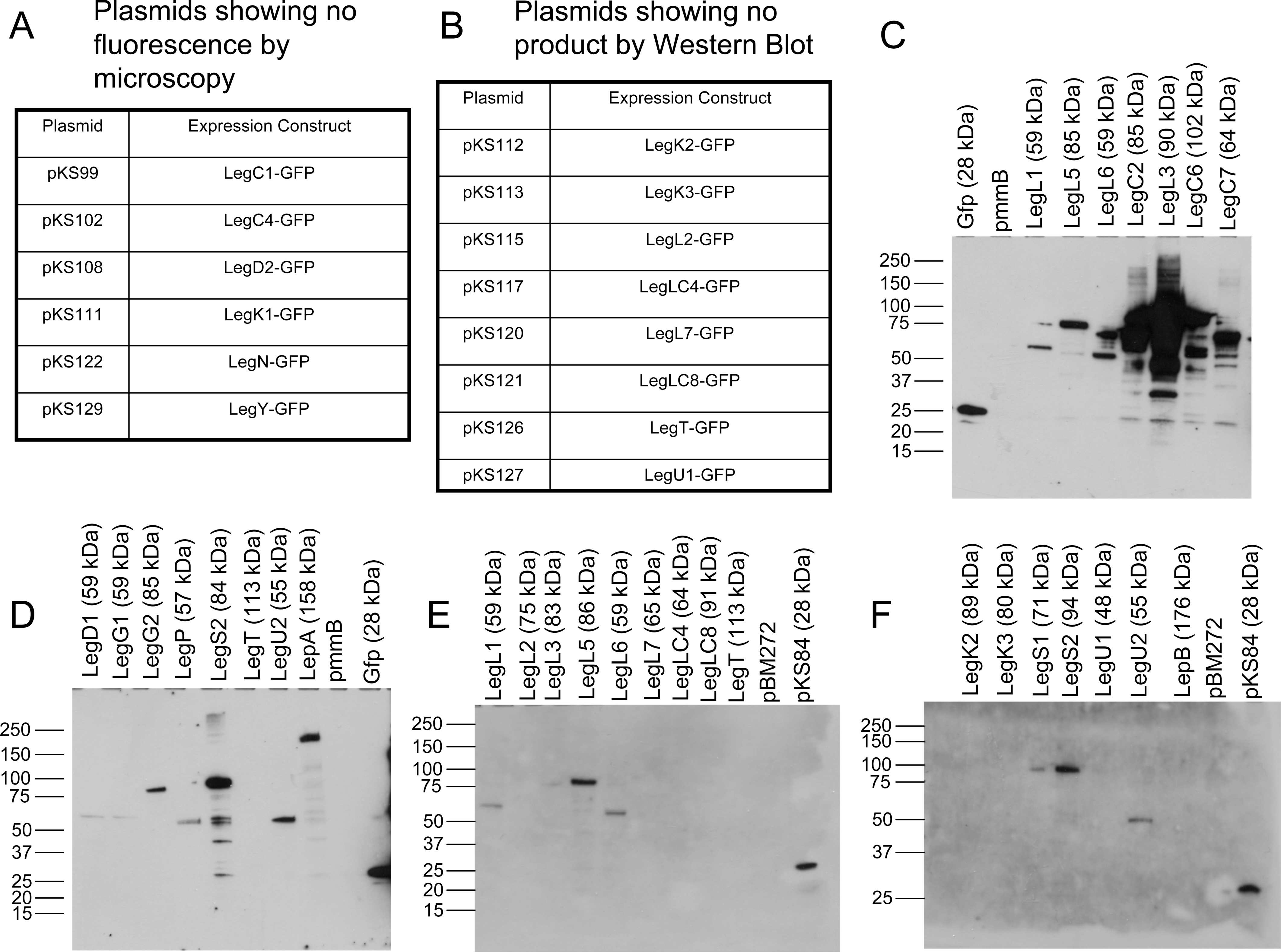

Supplement: Figure S1 — Detection of Leg-GFP proteins in yeast by immunoblot analysis. (A) Table A shows the plasmids that did not result in detectable fluorescence in yeast after galactose induction as determined by fluorescent microscopy. (B) Table B shows plasmids that did not produce a detectable band after immunoblotting but did show low levels or diffuse GFP localization by fluorescent microscopy. (C, D, E, F) Immunoblots on yeast lysates following galactose induction. The expression construct and predicted molecular weight are shown above each respective lane. Immunoblots were performed as described in Materials and Methods. (2.72 MB TIF) [file ppat.1000117.s001.tif]
